# Magnitude of Neonatal Jaundice and Its Associated Factor in Neonatal Intensive Care Units of Mekelle City Public Hospitals, Northern Ethiopia

**DOI:** 10.1155/2019/1054943

**Published:** 2019-04-10

**Authors:** Eyasu A. Lake, Gerezgiher B. Abera, Gedion A. Azeze, Natnaeal A. Gebeyew, Birhanu W. Demissie

**Affiliations:** ^1^College of Health Sciences and Medicine, Wolaita Sodo University, Sodo, Ethiopia; ^2^College of Health Sciences, Mekelle University, Mekelle, Ethiopia

## Abstract

**Background:**

Jaundice in the neonate is one of the most common clinical problems. Globally, every year about 1.1 million babies develop it and the vast majority reside in sub-Saharan Africa and South Asia. Study on magnitude and local factors associated with neonatal jaundice is limited in Ethiopia. So this study was aimed at assessing magnitude and predictors of neonatal jaundice among neonates admitted to neonatal intensive care unit of public hospitals in Mekelle city, Northern Ethiopia.

**Methods:**

Institution based cross-sectional study was conducted from February to April 2016 in neonatal intensive care unit of Mekelle city public hospitals. Systematic random sampling technique was used to select study participants. Data was collected by interviewing mothers through structured questionnaire and reviewing neonates' medical records using checklist. Multivariable binary logistic regression analyses were employed to identify factors associated with neonatal jaundice.

**Results:**

A total of 209 neonates with their mothers were included. The proportion of neonatal jaundice was found to be 37.3%. Prolonged labor [AOR = 4.39; 95% CI (1.8-10.69)], being male [AOR = 3.7; 95% CI (1.54-8.87)], maternal “O” blood group [AOR = 5.05; 95% CI (1.53-16.72)], sepsis [AOR = 2.64; 95% CI (1.15-6.05)], and blood type incompatibility [AOR = 18.21; 95% CI (6.36-52.13)] were positively associated with neonatal jaundice while night time delivery [AOR 0.42; 95% CI (0.18-0.96)] showed negative association.

**Conclusion:**

The magnitude of neonatal jaundice among neonates was found to be high. Duration of labor, time of delivery, sexes of neonate, sepsis, maternal blood group, and blood type incompatibility were significantly associated with neonatal jaundice. Therefore, improving newborn care and timely intervention for neonates with ABO/Rh incompatibility are recommended.

## 1. Background

Neonatal jaundice is yellowish discoloration of the skin, sclera, and mucous membranes due to accumulation of unconjugated, nonpolar, lipid-soluble bilirubin pigment in the skin [1, 2]. Jaundice in the new born is the most common clinical problem that requires close attention [3], evaluation, and treatment as it is the most common cause of neonatal readmission during neonatal period [4, 5]. It is among those easily preventable and treatable clinical conditions despite the fact that letting it in advance can lead to irreversible clinical problem that ends up with neonatal mortality.

Neonatal jaundice has a significant importance in neonatal morbidity and mortality world-wide [6–8]. It occurs in up to 60% [9] of term and 80% [3] of preterm newborns in the first week of life. About 10% of breast-fed babies are still suffering from neonatal jaundice during the first months of their life [4]. It is also accountable for 70% neonatal morbidity and 10% mortality [6, 10]. About 75% of neonatal mortality because of jaundice complication occurred in South Asia and sub-Saharan Africa [8].

In Ethiopia, neonatal mortality and morbidity are among the highest in the world, on which more than one-third of childhood death occurs within the first 28 days of age [11]. As studies revealed the incidence, etiology and risk factors of neonatal jaundice vary according to ethnicity, economic status, and geographical differences of countries [12]. But, there is limited data on the magnitude of neonatal jaundice in Ethiopia. So this study was aimed at assessing the magnitude of neonatal jaundice and its determinants in Mekelle city, Northern Ethiopia.

## 2. Methods and Materials

### 2.1. Study Design and Setting

An institution based cross-sectional study was conducted to assess the magnitude of neonatal jaundice and its associated factors at neonatal intensive care unit of Mekelle city public hospitals from February to April 2016. Mekelle is located 783kms north of Addis Ababa with a latitude and longitude of 13°29′N 39°28′E coordinates. Based on the 2007 Census conducted by the Central Statistical Agency of Ethiopia (CSA), the total population projection value of 2016 at Mekelle city is 340,858 of whom 168,261 are females [13].

Mekelle city has three public hospitals, i.e., Ayder referral hospital, Mekelle hospital, and Quiha hospital. Ayder referral hospital is one of the biggest tertiary level referral and teaching hospitals in Ethiopia which provides a broad range of medical services to both in and out patients of all age group in its catchment area [14]. Similarly, Mekelle hospital is the biggest regional hospital in Tigray region. Service of neonatal intensive care was rendered in Ayder and Mekelle hospitals but not in Quiha hospital. Therefore this study was conducted in neonatal intensive care unit (NICU) of Ayder and Mekelle hospitals.

### 2.2. Sample Size and Sampling Procedure

The source populations were all neonates with their mothers' who were admitted to neonatal intensive care unit of those hospitals. The sample size was determined by using single population proportion formula with the assumption of 35.0% [15] prevalence of neonatal jaundice, 95% confidence level, and 5% margin of error. The sample size was calculated to be 350, but since the estimated total populations in the study area during the study period were less than 10,000 the following correction formula was used:

(1) $$\begin{array}{c} nf=\frac{ni}{1+ni/N}=\frac{350}{1+350/416}=190 \end{array}$$

Assumptions

  nf= final sample size
  ni= initial sample size=350
  N=total estimated neonates with their mother in the study area during data collection time=416

BY adding 10 % nonresponses rate the final study participants were calculated to be 209. Study participants were selected by a systematic random sampling method. The number of neonates selected from each hospital was proportional to their case flow during the study period.

### 2.3. Exclusion Criteria

Neonate whose mother critically ill or unable to give informed consent.

Neonate who was admitted to NICU more than once during data collection time but either interviewed or excluded once by this study.

Late preterm neonates

### 2.4. Data Collection Procedures and Quality Control

The data were collected through interviewing of mother using structured questionnaire and review of medical records. The questionnaire was adapted from previous similar literatures [8, 15–17]. It had three parts; the first part was maternal sociodemographic characteristics, the second part was obstetric characteristics, and the third part embraces medical, environmental, and neonatal related factors.

Data was collected by four BSc nurses who had an experience of data collection and one day training was given. The overall supervision was carried out by the principal investigators. A pretest was conducted on 10% of similar study populations at Wukro hospital, located 47 km eastern of Mekelle city, and appropriate modifications were made.

### 2.5. Data Processing and Analysis

The filled questionnaires were checked for completeness and entered into EPI INFO version 3.5.3 statistical software and then exported to SPSS version 20 for further analysis. Descriptive statistics was made. Using bivariate analysis candidate variables were identified for multiple regressions at a p-value up to 0.25. Those eligible variables were entered into multiple logistic regression analysis to control the possible confounding variables and to identify independent predictors of neonatal jaundice. Finally, adjusted odds ratio (AOR) and their 95% CI were computed and variables with p-value less than 0.05 were considered as significantly associated with neonatal jaundice.

### 2.6. Diagnostic Criteria for Neonatal Jaundice

Gestational age is greater than or equal to 32wks.

*Neonatal Jaundice*. For this particular study, physician's diagnosis was used to identify neonatal jaundice. Then, since physicians did not register the type of neonatal jaundice as physiological, pathological, or kernicterus, those trained data collectors classified the type of jaundice using bilirubin level and IMNCI clinical features

### 2.7. Operational Definitions

*Neonate*. An infant from birth to 28 days of age.

*Late Preterm*. Newborn whose gestational age less than 32 weeks.

*Neonatal Jaundice*. Neonates diagnosed as jaundiced by physician.

*Physiological Jaundice*. Total bilirubin value along with IMNCI clinical features of physiological jaundice was used to diagnose physiological jaundice. Neonates in the presence of one more of the established IMNCI criteria (only skin on the face or eyes yellow and infant aged 2-13 days old) along with total bilirubin value 12mg/100ml in term babies and under 15mg/100ml in preterm babies [18, 19].

*Pathological Jaundice*. Total bilirubin value along with IMNCI clinical features of pathological jaundice was used to diagnose pathological jaundice. Neonates in the presence of one more the established IMNCI criteria (palms and/or soles yellow or skin and eyes yellow and baby is < 24 hrs old or skin and eyes yellow and baby is ≥14 days old) along with total bilirubin more than 12mg/100ml in term and more than 15mg/100ml in preterm [18, 19].

*Kernicterus*. It was defined on the basis of unconjugated hyperbilirubinaemia of more than 340*μ*mol/L in the term newborn or 200*μ*mol/L in preterm with features such as poor sucking, vomiting, drowsiness, hypertonia, paralysis of upward gaze, high pitched cry, involuntary movements, high fever, and convulsions in the established category [15].

*Skilled Birth Attendant*. A midwife, physician, nurse, or other health professionals who provide basic and emergency health care service to women and newborn during pregnancy, childbirth, and postpartum period.

*Neonatal Sepsis*. The hematological criteria along with the established Integrated Management of Neonatal and Childhood Illness (IMNCI) clinical features of neonatal sepsis were used to diagnose neonatal sepsis in this study. Neonates in the presence of one or more of the established IMNCI clinical features [either of fever (≥37.5°C) or hypothermia (≤ 35.5°C), fast breathing (≥60 breath per minute), severe chest indrawing, not feeding well, movement only when stimulated, convulsion, and lethargic or unconscious] along with ≥ 2 of the hematological criteria, total leukocyte count (<4000 or >12000 cells/m^3^, absolute neutrophil count (<1500 cells/mm3 or >7500 cells/mm^3^), erythrocyte sedimentation rate (ESR) (>15/1 h), and platelet count (<150 or >440 cells/m^3^) were considered to have neonatal sepsis [20].

*ABO Incompatibility*. For this particular study it was defined as neonates with laboratory confirmed to have ABO incompatibility [21].

## 3. Result

### 3.1. Sociodemographic Characteristic of Mothers

In this study a total of 209 neonates with their mother were included making 100% response rate. The median (±IQ range) age of mothers was 27±7 years ranging from 18 to 50 years, and age range of more than three-fourths of the mothers was between 20 and 35 years. About 142 (67.9%) mothers were living in urban areas. About 189 (90.4%) and 191 (91.4%) mothers were Tegaru in ethnicity and orthodox in religion, respectively. Of the total interviewed mothers, 90 (43.1%) were housewives by occupation.

Regarding educational status, unable to read and write (n=45), able to read and write (n=14), primary education (n=42), secondary education (n=49), and college and above (n=59) were documented among study participants.

Their marital status revealed that 197 (94.3%) mothers were married while 8 (3.8%) mothers were single. Regarding monthly income, 30(14.4%) mothers had a monthly income of 500 and below ETB, 76(36.4%) mothers had a monthly income of 500-1500 ETB, and the remaining 103 (49.3%) mothers had monthly income more than 1500 ETB [Table 1].

### 3.2. Neonate Characteristics

One hundred twenty-five (59.8%) neonates were male in sex and the median (±IQ range) of age at the time of admission was 1±3 days ranging from 1 to 26 days of whom 90% (188) of neonates age lies within a week. As well, about 103 (49.3%) neonates were delivered at term with birth weight of 2.5 kg and above. In this study, APGAR score of 7-10 was recorded by 149 (71.3%) neonates. Regarding the feeding status, 191 (91.3%) neonates fed breast milk of whom 184 (88%) were EBF. In this study the blood group of neonates was assessed, blood groups A” “B”, “AB”, “O”, and unknown were recorded by 43,36, 22, 58, and 50 respondents, respectively [Table 2].

### 3.3. Obstetric Characteristics

The median (+IQ range) of parity was 2± 3 ranging from 1 to 13 live births. One hundred and seventy-four (83.3%) neonates were singleton. One hundred ninety-six (93.8 %) respondents had antenatal care (ANC) follow up at least once during pregnancy, but 13(6.2%) had not. In this study, gestational diabetes mellitus by 2(6.7%), gestational hypertension by 11(36.7%), premature rapture of membrane (PROM) by 16(53.3%), and hyperemesis gravidarum by 1 (3.3%) mothers are identified as obstetric complication.

With regard to mode of delivery, spontaneous vaginal delivery accounted for 150 (71.8%) whereas instrumental and cesarean section 14 (6.7%) and 45 (21.5%) mothers, respectively. The duration of labor ranges from 4 to 72 hours of which normal duration of labor was recorded by 146 (69.9%) participants. Oxytocin was used as induction of labor by 97 (46.6%) mothers.

### 3.4. Environmental and Medical Factors

One hundred and thirty-one (62.7%) and 67 (32.1%) neonates were delivered at hospital and health center, respectively. Home delivery was accounted by 11(5.3%) mothers. Regarding birth attendant, 198 (94.7%) deliveries were attended by a skilled birth attendant, 10 (4.8%) by a traditional birth attendant, and 1 (0.5%) no attendant at all. About 113 (54.1%) neonates were delivered at day time.

About three-fourths 158 (75.6%) mothers took drugs during pregnancy, and insulin 2 (1.3%), iron with folic acid 145 (91.8%), magnesium sulphate 3 (1.9%), and others 8 (5.1%) were the drugs taken by respondents. Regarding the substance use, 22 (10.5%) mothers took substances during pregnancy of the participant neonate. The substances were alcohol, 19 (86.4%), chat 1 (4.5%), and herbal medication 2 (9.1%). In this study the blood group of mothers was assessed; blood groups “A” “B”, “AB”, “O”, and unknown were witnessed by 55, 46, 14, 43, and 51 respondents, respectively. Sepsis (n=98), ABO incompatibility (n= 29), and Rh incompatibility (n=22) were documented as a medical complication.

### 3.5. Proportion of Neonatal Jaundice

The proportion of neonatal jaundice among neonates admitted to the neonatal intensive care unit of Mekelle city public hospitals was found to be 37.3% (78). Among these 46 (22%) cases were pathological jaundice and the rest 32 (15%) were found to be physiological jaundice. All jaundiced neonates took phototherapy as a mode of treatment, but no exchange blood transfusion.

### 3.6. Bivariate Analysis of Different Factors with Neonatal Jaundice

On bivariate binary logistic regression, the odds neonatal jaundice among mothers with a monthly of ≤ 500 Ethiopian birr was 66% less likely compared to mothers with a monthly income of greater than 1500 Ethiopian birr [COR = 0.34; 95% CI (0.13-0.89)]. Neonates who were born at health center were 60% less likely to have neonatal jaundice compared to neonate who were born at hospital [COR = 0.4; 95% CI (0.2-0.76)].

Regarding time of delivery, night time delivery was 66% less risk for neonatal jaundice compared to day time delivery [COR = 0.33; 95 % CI (0.18-0.60)]. Prolonged duration of labor had 2.72 times higher risk for neonatal jaundice compared to normal duration of labor [COR = 2.72; 95% CI (1.46-4.99)].

Neonatal jaundice among male neonate was 4.68 times more likely compared to female neonates [COR = 4.68; 95% CI (2.42-9.04)]. Similarly neonates with “O” blood group were 2.38 timely more likely to have neonatal jaundice compared with those neonate with “A” blood group [COR = 2.38; 95% CI (1.05-5.4)].

The odds of neonatal jaundice among mothers with “O” blood group were 2.6 times more likely compared to those with “A” blood group [COR = 2.6; 95% CI (1.14-5.92)]. Septic neonates were 2.59 times higher risk for neonatal jaundice compared with nonseptic neonates [COR = 2.59; 95% CI (1.46-4.61)].

Moreover, neonate who had a family/sibling history of jaundice was 2.82 times more likely to develop neonatal jaundice compared to a neonate with no family/sibling history of jaundice [COR = 2.82; 95% CI (1.38-5.79)]. Blood incompatibility was 17.71 times higher risk for neonatal jaundice compared to a compatible blood group [COR = 17.71; 95% CI (7.34-42.77)] [Table 3].

### 3.7. Factors Associated with Neonatal Jaundice

Multivariable binary logistic regression analysis was done by taking variables showing significant association on bivariate analysis at p-value of ≤0.25 to control (adjust) the possible confounding. Prolonged duration of labor, time of delivery, mothers blood group, sex of neonate, sepsis, and blood incompatibility had a significant association with neonatal jaundice at p-value <0.05 in multivariate analysis.

The finding of this study showed that the odds of having neonatal jaundice among neonates who were delivered with long duration of labor were almost 4.4 times higher compared with those who were delivered with a normal duration of labor [AOR = 4.39; 95% CI (1.8-10.69)]. As well, neonates who were born during night time were 58% less likely to have odds of neonatal jaundice compared with those neonates who were born during day time [AOR = 0.42; 95% CI (0.18-0.96)],

The odd of neonatal jaundice among male neonates was 3.7 times higher compared with those female neonates [AOR = 3.7; 95% CI (1.54-8.87)]. Similarly, the odds of developing neonatal jaundice among neonates whose mother had “O” blood group were five times higher compared with those neonates whose mother had “A” blood group [AOR = 5.05; 95% CI (1.53-16.72)].

In this study sepsis and blood type incompatibility had significant association with the dependent variable. The odds of neonatal jaundice among neonates who had sepsis was 2.6 times higher compared with those neonates who had no sepsis diagnosis [AOR = 2.64; 95% CI (1.15-6.05)]. In the same way, neonates with blood type incompatibility had higher odds of neonatal jaundice compared with those neonates without blood type incompatibility [AOR = 18.21; 95% CI (6.36-52.13)] (Table 4).

## 4. Discussion

Neonatal jaundice has a significant importance on neonatal morbidity and a little bit on neonatal mortality world-wide. The vast majority of the affected neonates reside in sub-Saharan Africa and South Asia [15, 22]. Limited data were available on magnitude and local factors associated with neonatal jaundice in Ethiopia. This study was aimed at assessing proportion and predictors of neonatal jaundice among neonates admitted to neonatal intensive care unit of public hospitals in Mekelle city, Northern Ethiopia.

The proportion of neonatal jaundice was found to be 37.3%. This finding was consistent with previous study conducted in Southeast Nigeria (35 %) [15]. However, it was lower than finding from Shimla India (64%) [22]. On the other hand, this result was higher than the findings from Osijek Croatia (24.8 %) [23], Tehran (12.6 %) [5], Southern Nepal (2.93%) [16], Egypt (16.6%) [24], Lagos Nigeria (6.7%) [25], Benin City (26.5%) [17], Niger Delta University Teaching Hospital Nigeria (17.9%) [26], and Ethiopia (26.45%) [27]. The difference could be due to variation in study setting, time, and design among different studies. Besides, the variations might be due to difference in sociocultural and economic condition, level of obstetrics care, and gestational age among study populations.

This study had shown that prolonged duration of labor had significant effect on development of neonatal jaundice. The odd of jaundice was about four times higher among neonates who were born with long duration of labor compared with those neonates born in normal labor. This finding was in line with findings in Nepal [16]. This might be attributed to bruising and swelling of scalp of newborns due to the excessive pressure applied by birth attendants as a solution for prolonged labor in turn increases risk of jaundice by increasing bilirubin level in the blood [28].

Timing of delivery was significantly associated with neonatal jaundice with an increased risk observed among neonates born during day time. Neonates who were born during night time were 58% less likely to develop neonatal jaundice compared to those who were born during day time. This could be explained by the fact that room temperature is high during day time compared with night time. Considering the relative high room temperature during day time there might be improper immediate new born care especially on keeping the newborn warm that leads to hypothermia which is a known risk factor of neonatal jaundice by increasing unconjugated serum bilirubin level [29].

This study revealed that male neonates had higher odds of developing neonatal jaundice compared to their female counterparts. The finding was supported by studies done in Nepal [16] and Nigeria [25]. Conversely, this result was inconsistent with findings in Croatia [23], Iran [5], and Egypt [24]. This finding could explain that male newborns have relatively immature liver which may not be able to process all the bilirubin formed from red blood cells [30, 31].

It was also found that the odds of developing neonatal jaundice among neonates whose mother had “O” blood group were almost five times higher compared with those neonates whose mother had “A” blood group. Study done in Iran showed that mothers of “O” blood group had no significant effect on development of neonatal jaundice [5].

Moreover, the study revealed that neonatal jaundice had significant association with sepsis. The odds of developing neonatal jaundice among neonates who had sepsis were about two times higher compared with those neonates who had no sepsis diagnosis. Sepsis was also identified as the possible causes of neonatal jaundice in studies conducted in India [22, 32, 33], Iran [34, 35], and Nigeria [15, 26]. This might be due to the fact that sepsis might cause hemolysis of red blood cells and hepatic dysfunction that leads to accumulation of serum bilirubin in the body [36, 37].

Another contributing factor of neonatal jaundice was blood incompatibility. In this study 27 (93.1%) neonates with ABO incompatibility developed neonatal jaundice and it represents that 34.62% of the total jaundiced neonates had ABO incompatibility. Similarly, this disorder was reported as a leading cause of neonatal jaundice in the studies conducted in Canada [38], India [22, 33], Iran [35], Egypt [24], and Benin [17].

The limitation of the study was using small sample size; study participants with unknown ABO blood group were numbered as lacking ABO/Rh incompatibility; and G6PD was not assessed.

## 5. Conclusion

Neonatal jaundice is one of the common causes of neonatal morbidity in neonatal intensive care unit (NICU). The magnitude of neonatal jaundice among neonates admitted to neonatal intensive care unit of Mekelle city public hospitals was high. Being male, day time delivery, prolonged duration of labor, maternal “O” blood group, sepsis, and blood type incompatibility were the independent predictors of neonatal jaundice. It is recommended to have blood test during pregnancy with early intervention for those who had risks of fetomaternal blood group incompatibility.

## Figures and Tables

**Table 1:** Maternal Sociodemographic distribution of mothers at neonatal intensive care unit of Mekelle city public hospitals, Northern Ethiopia, 2016.

Variable	Category	Frequency	Percentage (%)
Mothers age	<20	13	6.2
	20-35	181	86.6
	>35	15	7.2
Marital status	Single	8	3.8
	Married	197	94.3
	Divorced	4	1.9
Residence	Urban	142	67.9
	Rural	67	32.1
Occupation	House wives	90	43.1
	Farmer	39	18.7
	Governmental employee	48	23.0
	Nongovernmental employee	12	5.7
	Merchant	20	9.6

**Table 2:** Neonatal characteristic distribution among neonates admitted to neonatal intensive care unit of Mekelle city public hospitals, Northern Ethiopia, 2016.

Characteristics	Category	Frequency	Percentage (%)
Sex of neonate	Male	125	59.8
	Female	84	40.2
Blood group	A	43	20.5
	B	36	17.3
	AB	22	10.5
	O	58	27.8
	Unknown	50	23.9
Gestational age at birth(in weeks)	<37	60	28.7
	37-42	143	68.4
	>42	6	2.9
Birth weight (kg)	<2.5	80	38.3
	≥2.5	129	61.7
Five minute APGAR score	≤6	60	28.7
	7-10	149	71.3
Family/sibling history of jaundice	Yes	38	18.2
	No	171	81.8

**(a):** 

Variable	Category	Jaundice		COR(95%CI)	P-value
		Yes	No		
		N (%)	N (%)		
Residence	Urban	57(40.1)	85(59.9)	1	
	Rural	21(31.3)	46(68.7)	0.68(0.37-1.26)	0.221
Maternal occupation	Housewife	34(37.8)	56(62.2)	1	
	Farmer	9(23.1)	30(76.9)	0.49(0.21-1.17)	0.107
	Governmental employee	24(50)	24(50)	1.65(0.81-3.34)	0.167
	Nongovernmental employee	4(33.3)	8(66.7)	0.82(0.23-2.94)	0.765
	Merchant	7(35)	13(65)	0.89(0.32-2.44)	0.816
Average monthly income	≤500	6(20)	24(80)	0.34(0.13-0.89)	0.028
	500-1500	28(36.8)	48(63.2)	0.78(0.43-1.44)	0.428
	>1500	44(42.7)	59(57.3)	1	
Types of pregnancy	Single	68(39.1)	106(60.9)	1	
	Multiple	10(28.6)	25(71.4)	0.62(0.28-1.38)	0.244
Place of delivery	Home	4(36.4)	7(63.6)	0.72(0.2-2.58)	0.613
	Health center	16(23.9)	51(76.1)	0.4(0.2-0.76)	0.006
	Hospital	58(44.3)	73(55.7)	1	
Mode of delivery	SVD	50(33.3)	100(66.7)	1	
	Instrumental	7(50)	7(50)	2(0. 67-6.02)	0.217
	C/S	21(44.3)	24(55.7)	1.75(0.89-3.44)	0.105

COR=crude odds ratio; SVD=spontaneous vaginal delivery; C/S=cesarean section.

**(b):** 

Variable	Category	Presence of Jaundice			
				COR(95%CI)	P-value
		Yes	NO		
		N (%)	N (%)		
Oxytocin during labor	Yes	46(47.4)	51(52.6)	2.26(1.27-3.99)	0.005
	No	32(28.6)	80(71.4)	1	
Duration of labor	Normal	44(30.1)	102(69.9)	1	
	Prolonged	34(54)	29(46)	2.72(1.46-4.99)	0.001
Timing of delivery	Day	55(51.3)	58(48.7)	0.33(0.18-0.60)	<0.001
	Night	23(24)	73(76)	1	
Sex of neonate	Male	63(50.4)	62(49.6)	4.68(2.42-9.04)	<0.001
	Female	15(17.9)	69(82.1)	1	
Gestational age	<37	25(41.7)	35(58.3)	1.37(0.74-2.54)	0.102
	37-42	49(34.3)	94(65.7)	1	
	>42	4(66.7)	2(33.3)	3.84(0.68-21.69)	0.56
Five minute APGAR score	≤6	27(45)	33(55)	1.57(0.85-2.896)	0.147
	7-10	51(34.2)	98(65.8)	1	
Neonates blood group	A	14(32.6)	29(67.4)	1	
	B	17(47.2)	19(52.8)	1.85(0.74-4.62)	0.186
	AB	7(31.8)	15(68.2)	0.97(0.32-2.91)	0.952
	O	31(53.4)	27(46.6)	2.38(1.05-5.4)	0.038
	Unknown	9(18)	41(82)	0.46(0.17-1.19)	0.109
Mothers blood group	A	18(32.7)	37(67.3)	1	
	B	22(47.8)	24(52.2)	1.88(0.84-4.23)	0.124
	AB	4(28.6)	10(71.4)	0.82(0.23-2.98)	0.766
	O	24(55.8)	19(44.2)	2.6(1.14-5.92)	0.023
	Unknown	10(19.6)	41(80.4)	0.50(0.21-1.22)	0.129
Sepsis	Yes	48(49)	50(51)	2.59(1.46-4.61)	0.001
	No	30(27)	81(73)	1	
Blood type incompatibility	Yes	39(84.8)	7(15.2)	17.71(7.34-42.77)	<0.001
	No	39(23.9)	124(76.1)	1	
Family/sibling history	Yes	22(57.9)	16(42.1)	2.82(1.38-5.79)	0.005
	No	56(32.7)	115(67.3)	1	

COR=crude odds ratio.

**Table 4:** Multivariate regression analysis of different variables with neonatal jaundice among neonates admitted to neonatal intensive care unit of Mekelle city public hospitals, Northern Ethiopia, 2016.

Variable	Category	Jaundice		Adjusted odds ratio (AOR) (95%CI)	p-value
		Yes	No		
Place of delivery	Home	4(36.4)	7(63.6)	4.37(0.8-23.79)	0.265
	Health center	16(23.9)	51(76.1)	0.58(0.2-1.69)	0.379
	Hospital	58(44.3)	73(55.7)	1	
Duration of labor	Normal	44(30.1)	102(69.9)	1	
	Prolonged	34(54)	29(46)	4.39(1.8-10.69)	0.010
Timing of delivery	Day	55(51.3)	58(48.7)	0.42(0.18-0.96)	0.039
	Night	23(24)	73(76)	1	
Sex of neonate	Male	63(50.4)	62(49.6)	3.7(1.54-8.87)	0.003
	Female	15(17.9)	69(82.1)	1	
Five minute APGAR score	≤6	27(45)	33(55)	1.06(0.41-2.75)	0.775
	7-10	51(34.2)	98(65.8)	1	
Neonates blood group	A	14(32.6)	29(67.4)	1	
	B	17(47.2)	19(52.8)	0.85(0.2-3.66)	0.697
	AB	7(31.8)	15(68.2)	0.53(0.1-2.78)	0.967
	O	31(53.4)	27(46.6)	1.7(0.56-5.16)	0.531
	Unknown	9(18)	41(82)	0.91(0.24-3.53)	0.170
Mothers blood group	A	18(32.7)	37(67.3)	1	
	B	22(47.8)	24(52.2)	2.66(0.82-8.61)	0.70
	AB	4(28.6)	10(71.4)	0.63(0.59-22.3)	0.751
	O	24(55.8)	19(44.2)	5.05(1.53-16.72)	0.012
	Unknown	10(19.6)	41(80.4)	0.93(0.29-3.00)	0.387
Sepsis	Yes	48(49)	50(51)	2.64(1.15-6.05)	0.022
	No	30(27)	81(73)	1	
Blood type incompatibility	Yes	39(84.8)	7(15.2)	18.21(6.36-52.13)	<0.001
	No	39(23.9)	124(76.1)	1	

*Note*. In bivariate analysis variables with P-value of ≤0.25 were entered in multivariate model.

## Data Availability

Data supporting this finding can be found upon request.

## Abbreviations

AOR: Adjusted odds ratio
ANC: Antenatal care
APGAR score: Appearance pulse grimace activity respirationscore
COR: Crude odds ratio
EDHS: Ethiopian Demographic and Health Survey
PNC: Postnatal care
NICU: Neonatal intensive care unit.
